# Non-invasive MR assessment of the microstructure and microcirculation in regional lymph nodes for rectal cancer: a study of intravoxel incoherent motion imaging

**DOI:** 10.1186/s40644-019-0255-z

**Published:** 2019-11-04

**Authors:** Xinyue Yang, Yan Chen, Ziqiang Wen, Yiyan Liu, Xiaojuan Xiao, Wen Liang, Shenping Yu

**Affiliations:** 1 Department of Radiology, Zhujiang Hospital of Southern Medical University, Guangzhou, Guangdong People’s Republic of China 510280; 2 Department of Radiology, The First Affiliated Hospital of Sun Yat-sen University, Guangzhou, Guangdong People’s Republic of China 510080; 3 Department of Radiology, The Eighth Affiliated Hospital of Sun Yat-sen University, Shenzhen, Guangdong People’s Republic of China 518033

**Keywords:** Intravoxel incoherent motion, MRI, Lymph nodes, Rectal cancer

## Abstract

**Background:**

The aim of this study is to evaluate the microstructure and microcirculation of regional lymph nodes (LNs) in rectal cancer by using non-invasive intravoxel incoherent motion MRI (IVIM-MRI), and to distinguish metastatic from non-metastatic LNs by quantitative parameters.

**Methods:**

All recruited patients underwent IVIM-MRI (*b* = 0, 5, 10, 20, 30, 40, 60, 80, 100, 150, 200, 400, 600, 1000, 1500 and 2000 s/mm^2^) on a 3.0 T MRI system. One hundred sixty-eight regional LNs with a short-axis diameter equal to or greater than 5 mm from 116 patients were evaluated by two radiologists independently, including 78 malignant LNs and 90 benign LNs. The following parameters were assessed: the short-axis diameter (S), long-axis diameter (L), short- to long-axis diameter ratio (S/L), pure diffusion coefficient (*D*), pseudo-diffusion coefficient (D^*^), and perfusion factor (*f*). Intraclass correlation coefficients (ICCs) were calculated to assess the interobserver agreement between two readers. Receiver operating characteristic curves were applied for analyzing statistically significant parameters.

**Results:**

Interobserver agreement of IVIM-MRI parameters between two readers was excellent (ICCs> 0.75). The metastatic group exhibited higher S, L and *D* (*P* < 0.001), but lower *f* (*P* < 0.001) than the non-metastatic group. The area under the curve (95% CI, sensitivity, specificity) of the multi-parameter combined equation for *D*, *f* and S was 0.811 (0.744~0.868, 62.82%, 87.78%). The diagnostic performance of the multi-parameter model was better than that of an individual parameter (*P* < 0.05).

**Conclusion:**

IVIM-MRI parameters provided information about the microstructure and microcirculation of regional LNs in rectal cancer, also improved diagnostic performance in identifying metastatic LNs.

## Background

The guidelines of the National Comprehensive Cancer Network (NCCN) emphasize the importance of neoadjuvant chemoradiotherapy for patients with rectal cancer [1]. Nodal status is a key point in determining the therapeutic strategy, which mainly depends on the TNM stage of the patient. In rectal cancer, for regional lymph nodes (LNs) with a short-axis diameter equal to or greater than 5 mm (S ≥ 5 mm), size is a commonly used criterion with considerable accuracy in discriminating malignant from benign LNs [2]. Although some morphological features, such as border contour, signal intensity, internal structure and chemical shift effects, improve diagnostic accuracy in the evaluation of nodal status, the utility of these features is still affected by the subjective judgement of different radiologists [2–4]. Morphological criteria are useful in clinical diagnoses, but the various quantitative parameters of functional magnetic resonance imaging (MRI) arouse scientific researchers’ interest because they provide more information about the tissue microenvironment than do morphological features.

Dynamic contrast-enhanced MRI (DCE-MRI) allows both the qualitative and quantitative assessment of the tissue microcirculation [5, 6]. Based on the “two-compartment model” given by Tofts et al., the quantitative parameters derived from DCE-MRI show the blood flow, vascular permeability and extravascular extracellular space (EES) of various tumours [7]. A previous study reported that the transfer constant (*K*^trans^) and fractional EES volume (*v*_e_) are helpful in distinguishing metastatic from non-metastatic LNs with sizes larger than 5 mm in rectal cancer [8]. However, DCE-MRI is not suitable for patients with renal inadequacy because it requires the intravenous injection of gadopentetate dimeglumine [7].

Diffusion-weighted imaging (DWI) quantifies the Brownian motion of water molecules in both the intracellular and extracellular compartments without the need to inject any contrast agent [9]. The apparent diffusion coefficient (ADC), a quantitative parameter derived from DWI, yields information about the microstructure of the cellular environment [9]. A previous study found that the ADC could be used to identify nodal status in rectal cancer [10], but another study showed that although DWI facilitated LN detection, the ADC could not reliably differentiate between malignant and benign LNs [11]. The inconsistency of these results might be attributable to the mono-exponential equation used to calculate the ADC values. In addition, the ADC includes a component originating from the blood flow in random microvessels [12], which can be considered an incoherent motion at the voxel level [13]. Thus, the ADC does not reflect the true molecular diffusion of water in a biological voxel.

Intravoxel incoherent motion (IVIM) is the microscopic translational motion in each MRI voxel and includes the molecular diffusion of water and microcirculation of blood in the capillary network [13]. IVIM-MRI acquires tissue diffusion and perfusion information simultaneously by fitting a bi-exponential equation to multiple *b*-value DWI data [13]. The quantitative parameters generated from IVIM-MRI include the pure diffusion coefficient (*D*), pseudo-diffusion coefficient (D^*^) and perfusion factor (*f*). These parameters allow inferences to be made about the microstructure and microcirculation of a biological voxel without the need to inject a contrast agent. The cutoff values and diagnostic accuracy of IVIM-MRI parameters in identifying nodal status in rectal cancer varies, as reported in a few previous studies [14, 15], and the usefulness of each parameter is inconsistent [14, 15].

In this prospective study, we sought potential information about the microstructure and microcirculation in LNs with S ≥ 5 mm from quantitative IVIM-MRI parameters and assessed whether these parameters are useful for predicting nodal status in rectal cancer.

## Methods

### Ethics statement

This study was approved by the medical ethics committee of the hospital, and written informed consent was obtained from each patient before participation.

### Patients

A total of 197 consecutive patients diagnosed with rectal carcinoma by endoscopic biopsy were recruited from January 2015 to August 2016. All patients with no history of pelvic surgery or contraindications to MR examination underwent preoperative MRI. Among these patients, 22 without scheduled curative surgery in our gastrointestinal department, as well as 50 receiving neoadjuvant chemoradiotherapy, were excluded. Nine patients were excluded because of poor MRI quality; the MR images of 6 patients had serious motion artefacts due to hip movements, and those of 3 patients had metal artefacts caused by titanium clips in the sigmoid colon or rectum. Thus, our prospective study enrolled 116 patients, 65 males and 51 females (mean age, 59.78 years, range 29–82 years). The time between MR examination and surgery was a maximum of 2 weeks (mean, 5 days; range, 1–14 days).

### IVIM-MRI protocols

A 3.0 Tesla MR system (Magnetom Verio, Siemens, Germany) with a 6-channel phased-array body coil was used for image acquisition. All patients were scanned in the supine position with a feet-first orientation. Rectal preparation was performed on the patients by pouring an appropriate amount (20–80 mL) of ultrasonic gel into the rectum, but this procedure was not performed if the tumours were large or located in the lower rectum. Unless contraindicated, 20 mg of raceanisodamine hydrochloride was injected intramuscularly 10–15 min before MR examination to reduce intestinal peristalsis and rectal spasm.

Pre-contrast axial, sagittal, coronal and oblique axial high-resolution turbo spin-echo T2-weighted images were obtained; the oblique axial planes were orthogonal to the tumour base. IVIM-MRI was performed in the axial orientation using a single-shot spin-echo echo-planar imaging pulse sequence prior to gadopentetate dimeglumine injection. A total of 16 *b* values (*b* = 0, 5, 10, 20, 30, 40, 60, 80, 100, 150, 200, 400, 600, 1000, 1500 and 2000 s/mm^2^) were applied to acquire the IVIM-MR images. In addition, fat-suppressed post-contrast T1WI and 3D-VIBE T1WI were acquired. The parameters for all the MRI protocols are shown in Table 1.

**Table 1:** MRI protocols for the imaging sequences

MRI Protocol	TR (ms)	TE (ms)	Slice Thickness (mm)	Slices	FOV (mm)	Voxel size (mm)	No. of Signals Acquired	Scanning time
Pre-contrast enhanced sequences								
Axial T2WI	3000	87	5.0	25	260 × 260	0.8 × 0.7 × 5.0	2	2′54”
Axial IVIM-MRI^a^	3800	74.4	6.0	21	300 × 300	2.7 × 2.7 × 6.0	2	6′1”
Sagittal T2WI	3000	87	3.0	19	180 × 180	0.7 × 0.6 × 3.0	2	2′30”
Coronal T2WI	4000	77	3.0	25	220 × 220	0.7 × 0.6 × 3.0	2	2′52”
Oblique axial T2WI	3000	84	3.0	24	180 × 180	0.6 × 0.6 × 3.0	2	3′18”
Post-contrast enhanced sequence								
Oblique axial fat-suppressed T1WI	716	12	3.0	18	180 × 180	0.8 × 0.6 × 3.0	2	3′41”
Coronal 3D-VIBE T1WI	10	4.9	1.0	144	380 × 380	1.0 × 1.0 × 1.0	1	3′10”

Note: ^a^, single-shot spin-echo echo-planar imaging, *b* = 0, 5, 10, 20, 30, 40, 60, 80, 100, 150, 200, 400, 600, 1000, 1500 and 2000 s/mm^2^

### Post-processing and data analyses

Based on the bi-exponential model introduced by Le Bihan et al. [13], the IVIM-MRI data were calculated using the following equation:

1 $$ {S}_b/{S}_0=\left(1-f\right)\kern0.5em \exp \kern0.5em \left(- bD\right)+f\;\exp\;\left(-{bD}^{\ast}\right) $$

where *S*_*b*_ is the signal intensity at the particular *b* value we used, *S*_0_ is the signal intensity for *b* = 0 s/mm^2^, *f* is the perfusion factor representing the fractional perfusion linked to the microcirculation, *D* is the pure diffusion coefficient, and *D*^∗^ represents perfusion-related diffusion or incoherent microcirculation and is named the pseudo-diffusion coefficient.

Post-processing of the IVIM-MRI data was performed by using a prototype software-based MATLAB algorithm (MATLAB Version 3.3; MathWorks, Natick, MA, USA). The maps of *D*, D^*^ and *f* were generated automatically in a voxel-by-voxel manner using the former 14 *b* values. A segmented fitting algorithm was used for more robust parametric estimation [16]. *D* and *f* were first estimated by linear least square fitting of the IVIM-MRI data with *b* ≥ 200 s/mm^2^, assuming that D^*^ was significantly greater than *D* so that the influence of pseudo-diffusion on signal decay was negligible for *b* ≥ 200 s/mm^2^. Then, D^*^ was estimated by applying the acquired *D* and *f* values to the equation with *b* < 200 s/mm^2^.

**Fig. 1:**
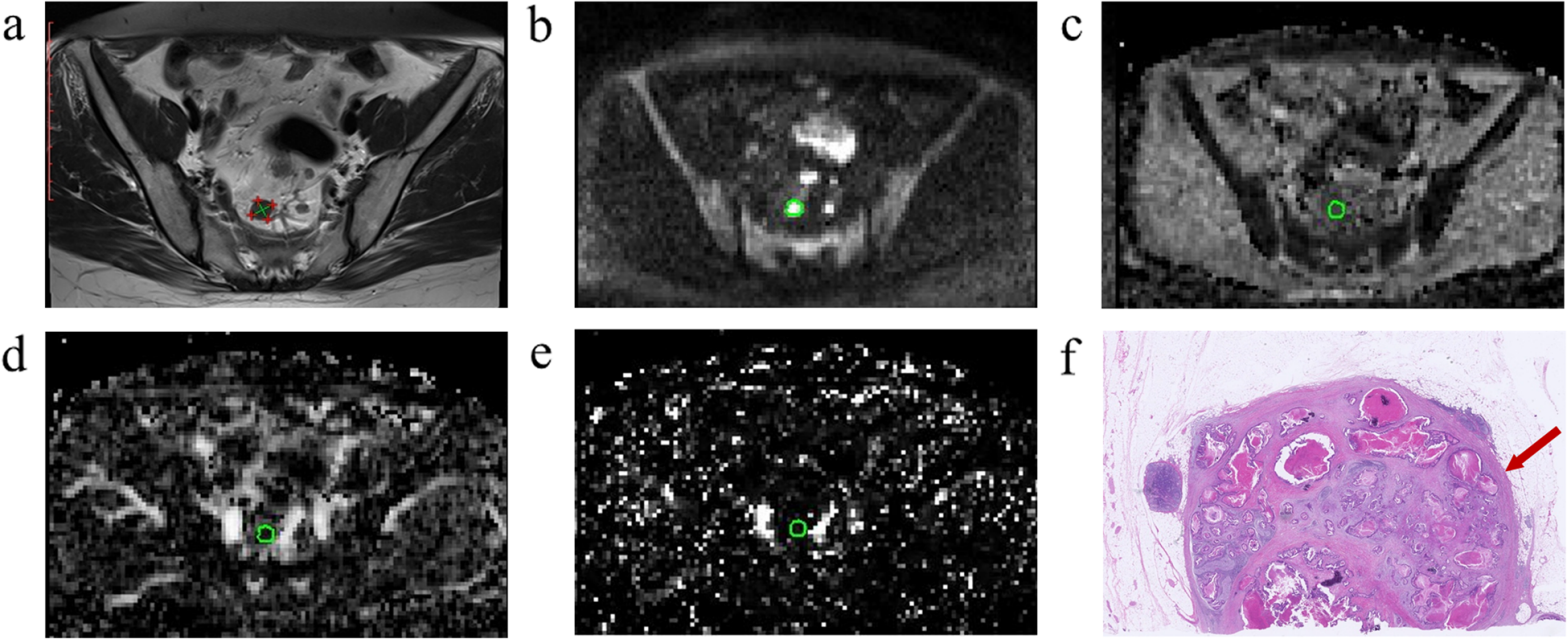
A 45-year-old female patient with stage IIIC (pT4aN2aM0) rectal cancer. **a** Choose a regional lymph node for post-processing. S (11.4 mm) and L (12.8 mm) are measured in the widest cross-section of this node, and S/L (0.89) is calculated. **b** A ROI is placed at the widest cross-section (green) on DWI map (*b* = 2000 s/mm^2^). **c, d, e** The ROI is copied to *D* map, D^*^ map, and *f* map respectively (green), then IVIM-DWI parameters are estimated (*D* = 0.656 × 10^−3^ mm^2^/s, D^*^ = 8.448 × 10^− 3^ mm^2^/s, *f* = 14.5%). **f** Metastatic adenocarcinoma is noted in this LN (red arrow) (hematoxylin-eosin stain, original magnification 40×)

IVIM-MR images were post-processed independently by two experienced radiologists blinded to the histopathologic findings. First, we measured the morphological parameters, including the short-axis diameter (S), long-axis diameter (L) and the short-to-long axis diameter ratio (S/L), of each LN in the field of view (FOV) on the widest cross-section of the T2W images (Fig. 1a). Then, only the regional LNs with S ≥ 5 mm in the FOV of the IVIM-MR images were post-processed. On the DW images with *b* = 2000 s/mm^2^, a region of interest (ROI) was drawn manually on the cross-section of a node by referring to the corresponding T2W images, covering the nodal parenchyma as much as possible and excluding visible necrosis and vessels. The median *D*, D^*^ and *f* of the ROI were estimated from the corresponding parametric maps [17]. The entire nodal volume was included by drawing ROIs for each individual slice, and the average values of each IVIM-MRI parameter over all ROIs were used for statistical comparison. An example of nodal post-processing is shown in Fig. 1b-e.

### Histopathologic assessment and nodal comparison

A node-for-node comparative scheme was constructed as follows by referring to previous articles [10, 18]. A surgeon with expertise in colorectal cancer and the radiologists who post-processed the IVIM-MRI images confirmed the nodal positions in the MR images. We divided regional LNs in the MR images into three groups, including the mesorectal, superior rectal and inferior mesenteric nodes. Then, based on the agreement regarding the nodal position, the regional LNs were successively localized, removed and numbered one by one in the three groups by the expert surgeon during the procedure. The nodes were promptly placed in individual trays and taken to the pathology department. All nodes were fixed in formalin and subsequently stained with haematoxylin-eosin (HE). A dedicated gastrointestinal pathologist classified the nodal status as metastatic or non-metastatic via light microscopic examination (Fig. 1f). Notably, other nodes that were not detected in the MR images were also harvested from the specimen and subjected to pathological examination to ensure that a sufficient number of LNs were pathologically examined [1].

According to the histopathologic findings, the radiologists re-matched the LNs with the preoperative IVIM-MR images in the corresponding groups, and a consensus was reached in cases of discrepancy between two readers. To ensure accurate node-for-node comparisons of the MR images and histopathologic findings, we devoted special attention to the nodal morphology in addition to the positions of the nodes relative to the tumour, rectal wall, mesorectal fascia, vessels, and adjacent nodes [10]. The LNs were excluded when this matching failed. Pathological staging of rectal cancer was performed in accordance with the American Joint Committee on Cancer (AJCC)/Union for International Cancer Control (UICC) guidelines for TNM staging [19].

### Statistics

Statistical analysis was performed using Statistical Package for the Social Sciences (SPSS, version 20.0, IBM, Inc., Chicago, IL) and MedCalc Statistical Software (version 18.2, Ostend, Belgium).

Interobserver agreement on each IVIM-MRI parameter for two independent readers was analysed by estimating the intraclass correlation coefficient (ICC). Based on the method given by Cicchetti [20], the ICC values were interpreted as follows: < 0.40, poor inter-rater agreement; 0.40–0.59, fair; 0.60–0.74, good; and 0.75–1.00, excellent. Bland-Altman plots were also constructed, and the limits of agreement (LoAs) were estimated from the plots.

According to the results of the one-sample Shapiro-Wilk test, continuous data were expressed as the means ± standard deviations (SDs) or the medians with interquartile ranges (IQRs). The independent-samples *t* test and the Mann-Whitney *U* test were applied for normally and non-normally distributed data, respectively.

Based on the results, we constructed receiver operation characteristic (ROC) curves for the statistically significant parameters derived from IVIM-MRI. Then, the optimum parameters were selected to establish a multi-parameter combined equation using the bi-logistic regression method. The ROC curve of predictive probability was also generated. The areas under the ROC curves (AUCs), with confidence intervals (CIs), were calculated. An AUC value of < 0.5 indicated no diagnostic performance; 0.5–0.7, poor performance; 0.7–0.9, moderate performance; and > 0.9, excellent performance. The maximum Youden indexes were used to determine the respective cutoff values producing the best diagnostic accuracy.

The chi-square test or Fisher’s exact test was used to compare the clinical and pathological features of rectal tumours between node-negative and node-positive patients. A *P* value of < 0.05 was considered statistically significant.

## Results

### Histopathologic findings

We harvested a total of 2089 LNs from the rectal specimens of 116 patients, with an average of 18 ± 10 nodes per patient. A total of 236 LNs from 49 patients contained metastases, and 1853 LNs were non-metastatic.

In the node-for-node evaluations, 245 LNs found on IVIM-MR images were matched exactly with LNs found on histopathologic examination, whereas 1844 nodes (132 malignant nodes and 1712 benign nodes) were not. Of the 245 matched LNs, 77 with a short-axis diameter of less than 5 mm were excluded from the evaluation. The remaining 168 LNs, including 78 malignant nodes and 90 benign nodes, were used for further analyses.

The clinical features and pathologic findings of the primary tumours versus LN status in the 116 patients are shown in Table 2.

**Table 2:** Clinical features and pathological findings of primary tumour versus lymph node status in 116 patients

parameter	total	lymph node status		*P*
		N^−^	N^+^	
Age (mean ± SD years)	59.78 ± 11.00	61.15 ± 10.80	57.92 ± 11.11	0.119^a^
Sex				0.191^b^
Male	65	34	31	
Female	51	33	18	
Primary tumour location				0.007^c^
Upper rectum	32	13	19	
Mid rectum	54	30	24	
Distal rectum	30	24	6	
Pathologic T staging				< 0.001^c^
Tis	2	2	0	
T1	5	5	0	
T2	27	22	5	
T3	31	20	11	
T4	51	18	33	
Histologic grade				< 0.001^c^
Well	10	10	0	
Moderate	69	55	14	
Poor	37	2	35	

^a^independent-samples *t* test

^b^Fisher exact test

^c^chi-square test

### Interobserver agreement

**Fig. 2:**
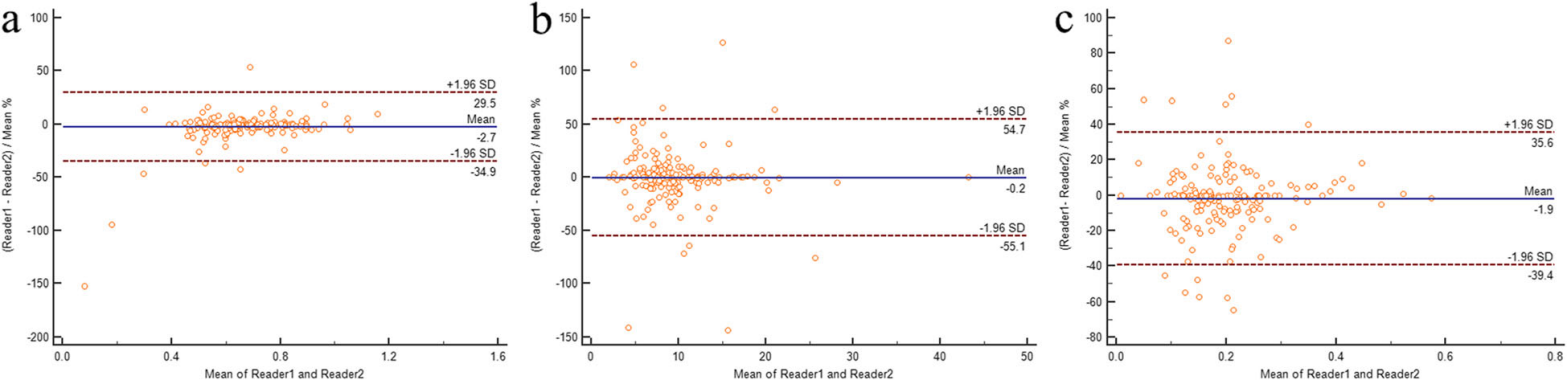
Bland-Altman plots of the *D* (**a**), D^*^ (**b**), *f* (**c**) measurements for 168 lymph nodes with short-axis diameter equal to or greater than 5 mm. X-axis is the average parametric value of two readers. Y-axis is the percentage difference in parametric value of two readers

The ICCs of the IVIM-MRI parameters reflected excellent interobserver agreement between the two independent readers (Table 3). The measured values of each parameter for all exclusive LNs are summarized in Table 3. The Bland-Altman plots in Fig. 2 show that the points in each plot tended to distribute around the mean difference line; most were within − 1.96 SD to + 1.96 SD of the mean.

**Table 3:** Interobserver agreement of quantitative parameters derived from IVIM-MRI between two readers for 168 lymph nodes with a short-axis diameter equal to or greater than 5 mm

Parameter	Reader1	Reader2	ICC	95% CI
*D* (10^−3^·mm^2^·s^−1^)	0.659 (0.216)	0.657 (0.210)	0.932	0.909~0.950
D^*^ (10^−3^·mm^2^·s^−1^)	8.385 (4.883)	8.473 (5.182)	0.804	0.743~0.852
*f*	0.182 (0.098)	0.186 (0.097)	0.916	0.888~0.937

Note: Value with number in parentheses is median with interquartile range; *ICC* intraclass correlation, *CI* confidence interval, *D* pure diffusion coefficient, D^*^ pseudo-diffusion coefficient, *f* perfusion factor

According to a previous study [17], we used only the measurement of the first reader for further analyses because the interobserver agreement for the IVIM-MRI parameters was excellent.

### Parametric comparison

The malignant LNs exhibited greater S, L and *D* values than the benign LNs (*P* < 0.001). However, a lower *f* was found for the malignant LNs than for the benign LNs (*P* < 0.001). S/L and D^*^ were not significantly different between the two groups (*P* = 0.582 and *P* = 0.126, respectively, Table 4).

**Table 4:** Quantitative parameters derived from IVIM-MRI versus histologic findings in 168 lymph nodes with a short-axis diameter equal to or greater than 5 mm

Parameter	Non-metastatic LNs	Metastatic LNs	*P*
	(*n* = 90)	(*n* = 78)	
S (mm)	6.085 (1.710)	6.650 (2.890)	< 0.001
L (mm)	6.835 (2.170)	7.850 (2.650)	< 0.001
S/L	0.864 (0.130)	0.865 (0.090)	0.582
*D*(10^−3^·mm^2^/s)	0.583 (0.183)	0.723 (0.213)	< 0.001
D^*^(10^−3^·mm^2^/s)	8.684 ± 4.245	7.798 ± 3.211	0.126
*f*	0.207 (0.133)	0.163 (0.079)	< 0.001

Note: Value with number in parentheses is median with interquartile range, value ± number is mean ± SD; *S* short-axis diameter, *L* long-axis diameter, *S/L* short- to long-axis diameter ratio, *D* pure diffusion coefficient, D^*^ pseudo-diffusion coefficient, *f* perfusion factor

### ROC curve analyses

The AUCs derived from the ROC curves of the quantitative parameters indicated that *D* exhibited moderate diagnostic performance for discriminating metastatic from non-metastatic LNs, but *f*, S and L exhibited low performance (Table 5). The cutoff values were 0.592 **×** 10^− 3^ mm^2^/s for *D*, 24.4% for *f*, 6.51 mm for S, and 6.87 mm for L. *D*, *f* and S were selected to establish the following multi-parameter combined equation based on the bi-logistic regression method:

**Table 5:** Diagnostic efficacy of ROC curves for 168 lymph nodes with a short-axis diameter equal to or more than 5 mm at the largest Youden index

Parameter	AUC(95% CI)	Youden index	Cutoff value	Sensitivity	Specificity
*D*	0.751 (0.679~0.815)	0.415	0.592 × 10^−3^ mm^2^/s	85.90%	55.56%
*f*	0.665 (0.589~0.736)	0.304	0.244	94.87%	35.56%
S	0.670 (0.594~0.741)	0.255	6.510 mm	57.69%	67.78%
L	0.676 (0.599~0.746)	0.303	6.870 mm	76.92%	53.33%
*D* + *f* + S	0.811 (0.744~0.868)	0.506	0.555	62.82%	87.78%

Note: *ROC* receiver operating characteristic, *AUC* area under curve, *S* short-axis diameter, *L* long-axis diameter, *D* pure diffusion coefficient, D^*^ pseudo-diffusion coefficient, *f* perfusion factor, *D* + *f* + S the multi-parameter model comprising *D*, *f* and short-axis diameter

2 $$ \log \kern0.5em P=-5.274+6.399{X}_1-6.577{X}_2+0.307{X}_3 $$

where *X*_1_ is the *D* value, *X*_2_ represents the *f* value, and *X*_3_ is the short-axis diameter.

**Fig. 3:**
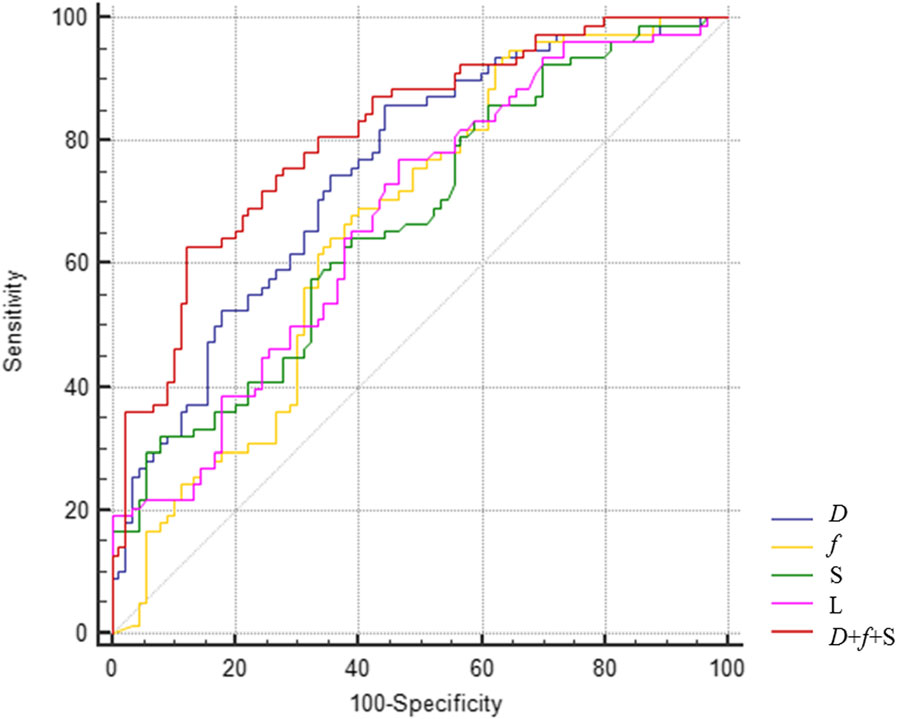
Receiver-operating characteristic (ROC) curves for the *D*, *f*, S, L, and multi-parameter model in discriminating between metastatic and non-metastatic regional lymph nodes with short-axis diameter equal to or greater than 5 mm in rectal cancer

The equation was indicated to be robust via the Hosmer-Lemeshow test (*P* > 0.1). The AUC (95% CI) of the ROC curve for logit *P* was 0.811 (0.744~0.868), which was greater than that of the other ROC curves when analysed via the pairwise comparison method (*P* < 0.05), thus demonstrating that the diagnostic performance of the combination of *D*, *f* and S was better than that of an individual parameter. Unfortunately, the AUCs of the ROC curves for the individual parameters were not significantly different (*P* > 0.05). The ROC curves and relative values are shown in Fig. 3 and Table 5, respectively.

## Discussion

Our study showed that the IVIM-MRI parameters exhibited excellent interobserver agreement between two independent readers, consistent with the results of a previous study [15]. The good reproducibility of parametric measurements might be attributable to the use of antispastic agents before MRI examination, which improves image quality and facilitates the manual drawing of ROIs. In our research, the S, L, and *D* values of the metastatic LNs were higher than those of the non-metastatic LNs, while the *f* value was lower. Although the malignant LNs showed a lower D^*^ than the benign LNs, we did not find a statistically significant difference between the two groups. The multi-parameter combined equation consisting of *D*, *f* and S improved diagnostic performance in defining nodal status.

Generally, tumour cell proliferation induces nodal enlargement. Although some published studies used size criteria to detect nodal metastasis in rectal cancer, the cutoff value for the short-axis diameter lacked consistency [2–4]. A pathological study indicated that positive LNs are usually larger than negative LNs, but there is considerable overlap between the two groups in rectal cancer [21]. Similarly, our study found higher S and L values in the metastatic group, but the diagnostic performance of these parameters in defining nodal involvement was poor. Thus, the size criterion might not be a reliable predictor for distinguishing malignant from benign LNs.

According to the bi-exponential equation given by Le Bihan et al., both the D^*^ and *f* parameters derived from IVIM-MRI are related to the microcirculation [13]. The perfusion factor *f* represents a volume fraction of water flowing in perfused capillaries, which mainly depends on the density of active capillaries [13]. We found that metastatic LNs exhibited a lower *f*, indicating a decrease in the density of active capillaries in positive nodes. We explained this result as follows. The blood supply of a LN is provided by one or more arterioles, which then branch into the capillary network [22, 23]. Capillaries empty into high endothelial venules (HEVs), where microvessels communicate with lymphatic vessels, and ultimately return to the hilar vein [22]. Neoangiogenesis is redundant for the growth of metastatic tumour cells because of the rich native vascularity of LNs [24]. Instead, capillary regression occurs as the result of tumour cell involvement [25]. Accordingly, *f* decreases in metastatic LNs, as we found. Our result was consistent with that of Yu et al. [14]. In addition, Wu et al. reported that metastatic LNs exhibit a lower *f* than non-metastatic LNs in cervical cancer [26]. However, the results of a study by Qiu et al. [15] contrasted with our results. This difference might be ascribed to various factors. Although *f* is mainly affected by relaxation effects and the T2 contribution [17, 27], the MR system, post-processing software, and *b* value distribution also influence the estimation of this parameter [15, 28].

Based on the IVIM theory, the pseudo-diffusion coefficient D^*^ is proportional to the mean capillary segment length and blood velocity, reflecting the blood volume flowing inside microvessels [13]. Because lymphatic vessels communicate with capillaries through HEVs [22], metastatic tumour cells invade the microcirculation and impede a part of the blood flow, thus resulting in decreased blood velocity. In addition, the mean capillary segment length becomes shorter because of capillary regression induced by tumour cell involvement. Thus, malignant LNs exhibit lower D^*^ values than benign LNs, which is corroborated by two of the abovementioned studies [14, 15]. Our study also showed lower D^*^ values in malignant LNs than in non-malignant LNs, but we did not find a statistically significant difference between the two groups, a result similar to that of the study by Wu et al. [26]. This lack of significance could be explained by the small number of included LNs, which might weaken the statistical results. However, some studies reported that positive LNs had higher D^*^ values than negative LNs [28, 29]. In the IVIM model, the parametric calculation is affected by the distribution of *b* values [15, 28]; the inclusion of a greater number of *b* values lower than 200 s/mm^2^ improves the characterization of the pseudo-diffusion effect [30, 31]. Although we used nine *b* values of less than 200 s/mm^2^, D^*^ exhibited the lowest ICC of the IVIM-MRI parameters, but its utility was still unclear. The utility of D^*^ might be limited by its high uncertainty and poor reproducibility, as previously reported [32–35].

*D* is the pure diffusion coefficient, which is related to tissue microstructure; it reflects the molecular diffusion of water in extracellular and intracellular spaces and the exchange between these two compartments [13]. Generally, *D* is inversely correlated with the cellularity and the cellular nucleus-to-cytoplasm ratio [14]. Because of the cellular polymorphism of malignancies, the intracellular space decreases in metastatic LNs [28]. However, as mentioned above, capillary regression and decreased blood volume result in necrosis inside metastatic nodes; this micronecrosis is invisible. Thus, the extracellular spaces of malignant LNs enlarge, inducing an increase in the molecular diffusion of water. This conclusion was corroborated by the increased *D* of metastatic LNs found in our study, which was concordant with the results of the study by Qiu et al. [15]. On the contrary, Yu et al. reported that malignant LNs exhibited a lower *D* [14]. The single-section ROI method is potentially limited by the inaccuracies resulting from the variation in ROI sizes and positions [17]. Our whole-node volume analysis involved entire LNs, which may minimize sampling bias, better capture the inherent intranodal heterogeneity and improve IVIM parameter assessment [17]. Various studies also reported a decreased *D* of metastatic LNs in breast cancer [28], lung cancer [36] and head and neck squamous cell carcinoma [29]. The inconsistency might be explained by the different histological types of the various tumours. In addition to cellularity, the stromal microenvironment, including the connective tissue fraction and interstitial fluid pressure, influences the molecular diffusion of water [26].

The multi-parameter model consisting of *D*, *f* and S showed moderate diagnostic performance in distinguishing metastatic from non-metastatic LNs and performed better than the individual parameters. Thus, we believe that IVIM-MRI parameters have potential value in defining nodal status because they can provide information about the microstructure and microcirculation. Based on the results, contrast-enhanced MRI using gadolinium should not be mandatory for every rectal cancer patient.

Some limitations of our study need to be carefully considered. First, we excluded LNs with a short-axis diameter of less than 5 mm because the smaller number of pixels makes these LNs susceptible to contamination by misregistration effects or partial volume effects from adjacent tissues. However, approximately 50% of malignant LNs in rectal cancer are smaller than 5 mm in diameter. Second, we did not analyse different histopathologic types of metastatic LNs in rectal cancer, for example, mucinous versus nonmucinous carcinoma. Third, we did not evaluate the effects of neoadjuvant chemoradiotherapy on the diffusion and perfusion parameters of LNs. Fourth, the distribution of *b* values lower than 200 s/mm^2^ requires further study.

## Conclusion

In conclusion, the *D* and *f* values derived from IVIM-MRI differed significantly between metastatic and non-metastatic LNs in rectal cancer. The diffusion and perfusion parameters provided additional information about the microstructure and microcirculation. Thus, the multi-parameter model comprising *D*, *f* and S exhibited improved performance in diagnosing nodal status.

## Data Availability

Please contact authors for data requests.

## Abbreviations

ADC: Apparent diffusion coefficient
AJCC: American Joint Committee on Cancer
AUC: Area under the curve
CI: Confidence interval
*D*: pure diffusion coefficient
*D*^*^: pseudo-diffusion coefficient
DCE: Dynamic contrast-enhanced
DWI: Diffusion-weighted imaging
EES: Extravascular extracellular space
*f*: perfusion factor
FOV: Field of view
HE: Haematoxylin-eosin
ICC: Intraclass correlation coefficient
IQR: Interquartile ranges
IVIM: Intravoxel incoherent motion
*K*^trans^: transfer constant
L: Long-axis diameter
LN: Lymph node
LoA: Limit of agreement
MRI: Magnetic resonance imaging
NCCN: National Comprehensive Cancer Network
ROC: Receiver operating characteristic
ROI: Region of interest
S: Short-axis diameter
S/L: Short- to long-axis diameter ratio
SD: Standard deviations
UICC: Union for International Cancer Control
*v*_e_: extravascular extracellular space volume
